# Bivalve Shell Utilization by Juvenile *Octopus vulgaris* in Sandy Substrates

**DOI:** 10.1002/ece3.71060

**Published:** 2025-05-15

**Authors:** Jorge Hernández‐Urcera, Samuel E. Soule, Miguel Cabanellas‐Reboredo, Ángel F. González

**Affiliations:** ^1^ ECOBIOMAR Research Group Institute of Marine Research (IIM‐CSIC) Vigo Spain; ^2^ Centro Oceanográfico de Illes Balears (COB‐IEO) CSIC Palma de Mallorca Balearic Islands Spain

**Keywords:** bivalve shells, camouflage, juvenile behavior, *Octopus vulgaris*, sandy habitats, shelter use

## Abstract

The early life stages of *Octopus vulgaris* face significant challenges in sandy environments, where shelter is limited and predation risk is high. This study examines how juvenile octopuses adapt to these conditions, focusing on their use of empty bivalve shells as shelters. Between May 2022 and June 2023, through four SCUBA diving expeditions in the Cíes Islands, NW Spain, nine juvenile octopuses were found inhabiting bivalve shells. The juveniles preferentially selected shells that fit their body size, providing protection and minimizing exposure. They manipulated the shells for security and positioned themselves strategically for optimal visual surveillance. Additionally, they used camouflage techniques, blending with the sandy substrate. When threatened, some octopuses displayed bipedal locomotion to enhance their escape. This study highlights the adaptive flexibility of juvenile *O. vulgaris* in response to predation pressures in sandy habitats, adding to the growing but limited knowledge of habitat‐specific behaviors that can help contribute to the conservation strategies of wild populations.

## Graphical abstract

This study investigates how juvenile *Octopus vulgaris* adapt to sandy environments with limited shelter and high predation risk by using empty bivalve shells for protection. Through four SCUBA expeditions in NW Spain, nine juveniles were observed using shells that fit their body size, strategically positioning themselves for surveillance and employing camouflage. These behaviors emphasize the species' adaptability and have important implications for conservation in sandy habitats.

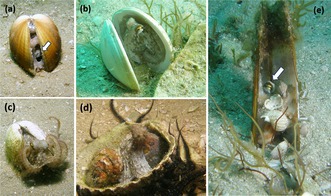

## 1 Introduction

The common octopus, *Octopus vulgaris* , is a highly adaptable species that thrives across various biotopes, including sandy and muddy bottoms, rocky areas, coral reefs, and seagrass beds (Mangold 1983). As an opportunistic carnivorous predator, adult *O. vulgaris* primarily feeds on crabs, mollusks, polychaetes, and bony fish (Mangold 1983). These octopuses modify their soft habitats by excavating dens and creating midden piles of prey remains (Mather 1991). The species is also known for its ability to drill holes in mollusk shells for feeding, a behavior observed even in juveniles (Guerra and Nixon 1987; Mather and Nixon 1990; Fee et al. 2023). To minimize predation risk and competition, *O. vulgaris* typically selects shelters, especially during daylight hours (Mather 1988). The characteristics of the substrate are thought to be a major factor influencing their density and distribution (Aronson 1991; Leite et al. 2009; Guerra et al. 2014).

Despite the extensive research on *O. vulgaris* as a species (Hanlon and Messenger 2018), there is a notable scarcity of information regarding the ecology of its juvenile stages in situ; partly due to the difficulty of finding juveniles in the wild. Most studies have focused on the adult population (Katsanevakis and Verriopoulos 2004a, 2004b; Guerra et al. 2015; Garci et al. 2016; Salvador et al. 2024), leaving a significant gap in our understanding of the behavior, survival strategy, and habitat preference of juveniles in their natural environment. Further, of the studies conducted on juveniles, many are focused on individuals reared in laboratories, which may limit the ability to provide environmentally relevant life history data (André et al. 2008; Hamlett 2024; Vergara‐Ovalle et al. 2024). This lack of data hinders the development of effective conservation strategies, as the early life stages are critical for the species' recruitment and population dynamics.

The early life stages of *O. vulgaris* are characterized by considerable vulnerability, particularly in sandy environments where they lack the shelter provided by more structurally complex habitats (Mather and O'Dor 1991). Juvenile octopuses are highly exposed to predators in these open spaces (Mather 1991), including conspecifics (Hernández‐Urcera et al. 2014, 2019), necessitating the development of specific survival strategies. Octopuses display notable behavioral flexibility and innovative problem‐solving skills with specific behavioral variation resulting from geomorphological factors (Fiorito et al. 1991; Villanueva et al. 2017; Dissegna et al. 2023). Additionally, they can modify the use of shelters based on the presence and type of predators and prey (Mather 1994). Adult specimens of *O. vulgaris* inhabiting sandy bottoms often excavate dens lined with shells of bivalve mollusks, providing refuge from predators (Katsanevakis and Verriopoulos 2004a; Guerra et al. 2014). However, for juvenile octopuses, this strategy may increase exposure to predators due to the time and activity required for den construction, in addition to its high energy cost. The observations presented in this work showed some behaviors and adaptive techniques employed by juvenile *O. vulgaris* to mitigate predation risk in sandy bottoms, with a particular focus on the utilization of empty bivalve shells as makeshift shelters.

## 2 Material and Methods

Between May 2022 and June 2023, a total of eight dives were conducted over four SCUBA diving expeditions in the sandy bottoms of the Cíes Islands (42°13′ 23.52" N, 8°53′ 56.61" W), located within the waters of the Atlantic Islands of Galicia National Park (NW Spain). These dives aimed to search for juvenile octopuses as part of the ECOSUMA project, whose primary objective was to enhance the understanding of the ecology of the common octopus during its early life stages. The dive site was chosen based on the findings of Guerra et al. (2014), who reported the presence of juvenile octopuses in the same sandy region. The dives were conducted by four experienced divers swimming in parallel while maintaining visual contact with each other. Belt transects were conducted between depths of 5 and 10 m, with each dive lasting approximately 70 min. During the dives, the divers stayed as close to the seabed as possible (between 15 and 30 cm) and inspected all the empty shells they encountered. Once a juvenile octopus was located, photographs were taken to record behaviors using an Olympus SP‐560UZ compact camera housed in an Olympus PT‐037 underwater housing, supported by an INON Z‐240 underwater strobe. When possible, the mantle length of the specimens was estimated using the shell size as a reference, with measurements taken using the software Fiji (Schindelin et al. 2012). The size of each bivalve shell was estimated based on the average size of each bivalve species in the study area (Trigo et al. 2018).

## 3 Results

**FIGURE 1:**
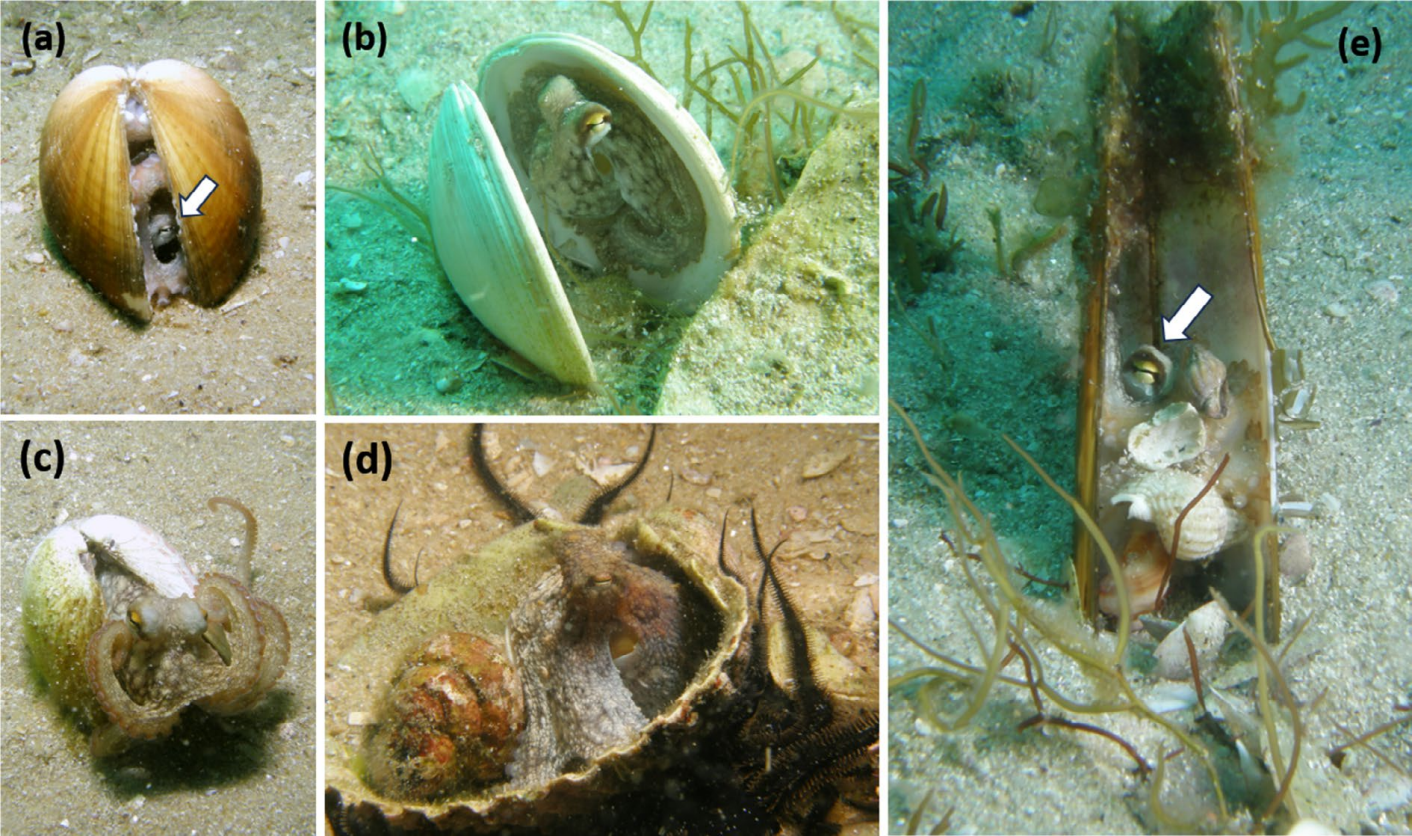
Juvenile octopuses ( *Octopus vulgaris* ) photographed in sandy bottoms off the Cíes Islands (NW Spain) using bivalve shells as shelters. The white arrows indicate the position of the octopus's eye. (a) Octopus hidden inside clam shells ( *Laevicardium crassum* ) with the shells almost closed. (b) Octopus hidden inside clam shells ( *Dosinia exoleta* ) with the shells separated. (c) Octopus attempting to emerge from the clam shell ( *Dosinia exoleta* ). (d) Octopus camouflaged inside a spiny cockle shell ( *Acanthocardia aculeata* ). (e) Octopus hidden inside razor clam shells (*Ensis* sp.) with the shells separated.

After completing the dives, a total of nine juvenile octopuses were found. The estimated mantle length of the octopuses ranged from 33 to 42 mm. All of these individuals were associated with empty bivalve mollusk shells (Figure 1). Six of them were found inside clam shells ( *Laevicardium crassum* , *Dosinia exoleta* , and *Ensis* sp.), while three of them, larger in size than the others, were located within spiny cockle shells ( *Acanthocardia aculeata* ). In all cases, the sizes of the shells were fairly similar to the size of the octopuses dwelling within them. Furthermore, in five of the six cases where octopuses were inside two‐valve shells, the shells were positioned with the valve opening oriented vertically.

In seven of the nine encounters, the juveniles remained inside the shells while the divers captured photographs (Figure 1a,b,d,e). If the divers approached too closely, the octopuses closed the valves of the shells. As the divers retreated, the octopuses reopened the valves or, in two cases, attempted to emerge from the shells before quickly retracting back inside (Figure 1c). Notably, two octopuses exhibited a unique method of hiding inside razor clam shells, positioning the empty shells vertically while holding the razor clam valves. Moreover, they used small gastropod shells, possibly not only for camouflage but also for protection, with only the octopus's eye being discernible to the divers (Figure 1e). In addition, octopuses that were more exposed, with shells opened wider, exhibited coloration resembling either the shells themselves (Figure 1b,e) or other elements present inside the shell, such as gastropod shells (Figure 1d).

**FIGURE 2:**
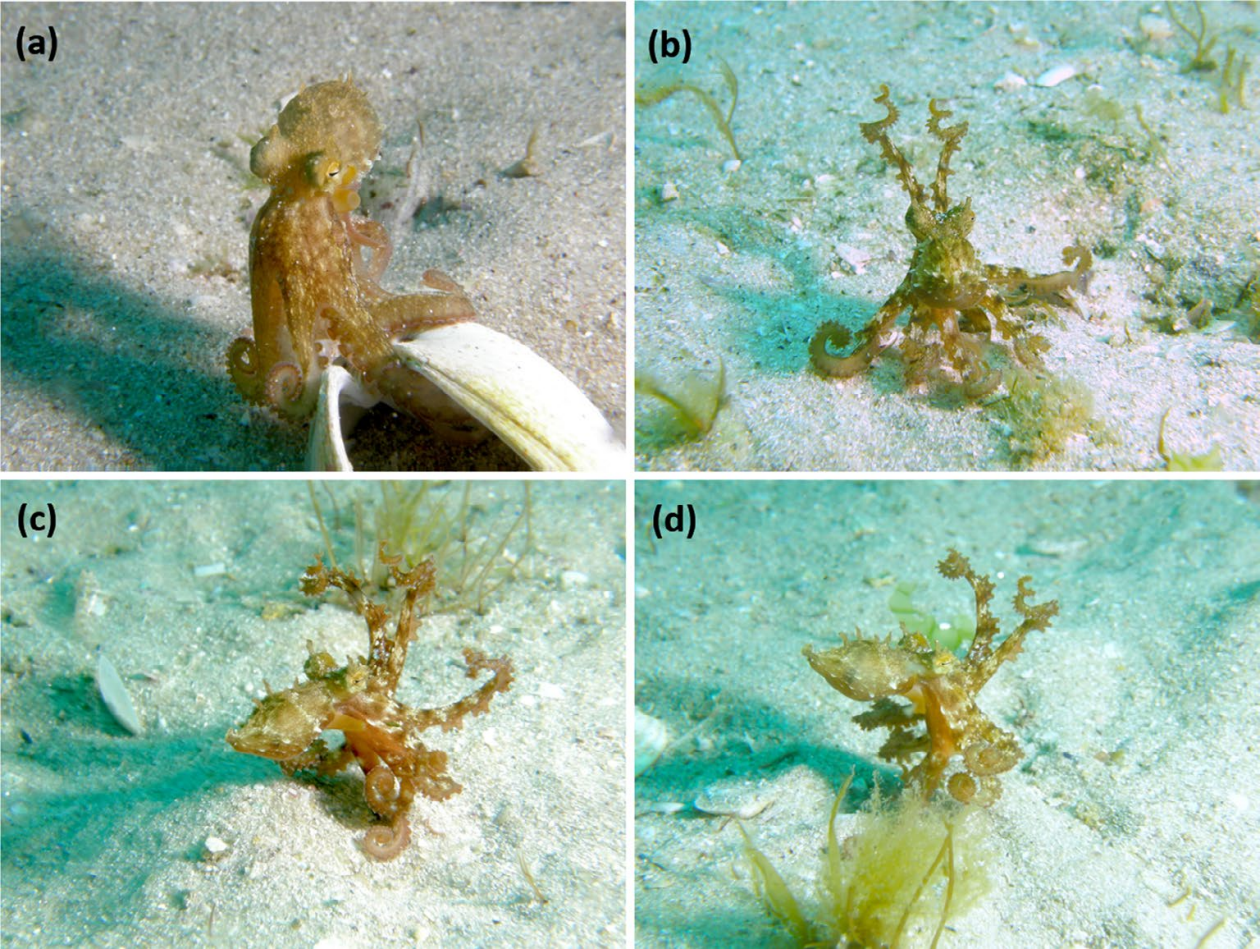
Juvenile octopus ( *Octopus vulgaris* ) abandoning its shelter (clam shell; *Dosinia exoleta* ) and moving away from the divers. (a) Octopus emerging from inside the clam shell. (b) Octopus showing a cryptic display, raising two of its arms above its head while sitting on the remaining 6 arms. (c) Octopus initiating bipedal locomotion. (d) Octopus finishing bipedal locomotion just before starting to swim by jet propulsion.

In two occasions, the octopuses completely left their shelter (Figure 2a). While they moved away, both octopuses exhibited bipedal locomotion before starting to swim by jet propulsion. Walking was preceded by a cryptic display in which the octopus coiled and raised the two front arms above its head while sitting on the remaining six (Figure 2b). Throughout the bipedal locomotion, the juveniles remained camouflaged (Figure 2c,d) raising both dorsal arms while using the right and left ventral arms to move.

## 4 Discussion and Conclusions

Juvenile octopuses in sandy habitats exhibited several adaptive behaviors that enhance their survival, illustrating how environmental diversity influences the development of learning and plasticity among these species. First, they demonstrate a keen ability to identify and occupy available shelters. In the absence of natural rock crevices or complex vegetation, bivalve shells become crucial for their survival (Guerra et al. 2014). The selection of bivalve shells was not random but appears to be influenced by the size and shape of the available shells. Juvenile octopuses preferred shells that closely fit their body size, providing a snug refuge that minimizes exposure. This selective behavior may suggest a level of cognitive assessment (Schnell et al. 2021), where the juveniles weigh the protective benefits of a shell against the potential risks of leaving a current shelter to find a better one.

The juvenile octopuses were able to manipulate the shells to create a more secure enclosure, often pulling the shell halves to form a more complete protective barrier. This ability to modify dens has been previously reported by Mather (1994) in rocky habitats in Bermuda, where juvenile octopuses modified dens by removing rocks from the interior and bringing items to block the den entrance. As octopuses inhabit a wide range of marine habitats, studies have pointed to the impact of these diverse ecological factors on the complexity of central nervous systems and visual pathways (Chung et al. 2022), potentially leading to specific behavioral variations. This variation is exhibited in our observations as the octopuses showed a remarkable feature when occupying the shells, ensuring they maintain a wide field of vision, indicative of heightened vigilance towards potential predators or prey. By positioning themselves within the shells, often with the shell aperture aligned vertically, these octopuses strategically optimize their visual surveillance capabilities. This positioning allows them to extend their sensory awareness beyond the confines of the shell, enabling rapid detection and response to environmental stimuli.

The juvenile octopuses also utilized their camouflage capability. The ability to effectively camouflage within these environments is another critical survival skill. *O. vulgaris* possesses advanced chromatophore systems that allow them to blend into the sandy substrate, making them less visible to predators (Messenger 1978). This dynamic camouflage was often complemented by the strategic placement of the bivalve shells, which can further obscure the octopus from potential threats and allow for more diverse color matching, as octopuses base their body patterns on selected features of nearby objects such as rocks, shells, or substrates (Josef et al. 2012). Moreover, when the octopuses left their lairs, they immediately displayed a characteristic camouflage, which they maintained as they moved away from the divers by bipedal locomotion. This type of behavior is consistent with that reported by Hernández‐Urcera et al. (2020), also observed on sandy bottoms of the Cíes Islands.

In conclusion, the survival of juvenile *O. vulgaris* in sandy habitats is heavily reliant on their ability to adapt and innovate in the face of predation threats. The use of empty bivalve shells as temporary refuges and the modifications of these “dens” with gastropod shells highlight their resourcefulness and underscore the importance of understanding habitat‐specific behaviors for conservation efforts. As octopuses inhabit a variety of ecological conditions, and early life stages are critical for their recruitment and population dynamics, future research should explore the cognitive and behavioral adaptations of juveniles. This will provide deeper insights into their survival mechanisms and inform conservation strategies to support their populations in varying marine environments.

## Author Contributions

**Jorge Hernández‐Urcera:** conceptualization (lead), data curation (lead), formal analysis (lead), investigation (equal), methodology (equal), writing – original draft (lead), writing – review and editing (equal). **Samuel E. Soule:** conceptualization (equal), investigation (equal), methodology (equal), writing – review and editing (equal). **Miguel Cabanellas‐Reboredo:** conceptualization (equal), investigation (equal), methodology (equal), writing – review and editing (equal). **Ángel F. González:** conceptualization (equal), funding acquisition (lead), investigation (equal), project administration (equal), writing – review and editing (equal).

## Ethics Statement

No animal testing was performed during this study.

## Conflicts of Interest

The authors declare no conflicts of interest.

## Data Availability

The authors have nothing to report.
